# Job Retention and Reintegration in People with Mental Health Problems: A Descriptive Evaluation of Supported Employment Routine Programs

**DOI:** 10.1007/s10488-022-01227-w

**Published:** 2022-10-26

**Authors:** Simeon J Zürcher, Micha Zürcher, Michael Burkhalter, Dirk Richter

**Affiliations:** 1 Center for Psychiatric Rehabilitation, Universitäre Psychiatrische Dienste Bern (UPD), Murtenstrasse 46, 3008 Bern, Switzerland; 2 University Hospital of Psychiatry and Psychotherapy, University of Bern, Bern, Switzerland; 3 Department of Health Professions, Bern University of Applied Sciences, Stadtbachstrasse 64, 3012 Bern, Switzerland

**Keywords:** job maintenance, job retention, mental illness, return to work, rehabilitation, reintegration, supported employment (SE)

## Abstract

**Purpose:**

Striking evidence supports the effectiveness of supported employment (SE) in achieving competitive employment in individuals with mental health problems. Yet, little is known whether SE is effective to maintain employment in individuals at risk of job loss. We aimed to descriptively compare SE for employed clients (SE-retention) and unemployed clients (SE-integration) regarding competitive employment.

**Methods:**

We used administrative data from January 2017 to October 2021 provided by a vocational rehabilitation center in Switzerland including all individuals (≥ 18yrs.) with mental health problems who participated either in SE-retention or SE-reintegration. The outcome was the proportion with competitive employment at discharge. Logistic regression was used to assess time trends and to descriptively compare SE-treatments. We used propensity score weighting, including personal, clinical and program-specific information to reduce group differences.

**Results:**

A total of 556 participants primarily diagnosed with mood/stress-related, schizophrenia and personality disorders were included (n **=** 297 SE-retention, n = 259 SE-reintegration) with median age 41 years and 57% female gender. The overall weighted comparison favored SE-retention over SE-reintegration OR 4.85 (95%-CI 3.10 to 7.58, p < 0.001) with predicted employment of 67.3% and 29.9% for SE-retention and SE-reintegration, respectively. While success for SE-reintegration remained stable over time, SE-retention showed an increase in more recent years.

**Conclusion:**

SE-retention provides an approach for early work-related support that can prevent labor market exclusion. In contrast, reintegration is likely to require more efforts to achieve employment and may result in less favorable outcomes. It is therefore necessary that further research includes appropriate comparison groups to evaluate the effectiveness of SE-retention programs as well as the economic and individual benefits.

**Supplementary Information:**

The online version contains supplementary material available at 10.1007/s10488-022-01227-w.

## Introduction

Mental ill-health is among the top reasons for sick leave, job loss and unemployment resulting in significant costs for the economy and the healthcare sector (OECD, 2012). Although, the majority of individuals with mental health problems (hereinafter defined as psychiatric/psychological symptoms and mental illness/disorders) desire to work, they are often excluded from the labor market which exposes them to the risk of further health decline, social exclusion and poverty (Gühne et al., 2021; Luciano & Meara, 2014; Richter & Hoffmann, 2019b). As an important cornerstone of social inclusion, work promotes mental health, mitigates clinical symptoms, is associated with improved quality of life, and enables individuals to live a self-determined life (Modini et al., 2016; Schuring et al., 2017). Aside from individual benefits, fostering job maintenance and reintegration can provide societal and economic advantages such as the reduction of disability benefits costs, availability of work force, and the reduction of organizational productivity loss.

Supported employment (SE) is a well-established and evidence based program to achieve competitive employment in people with mental health problems. With the approach of directly placing individuals into competitive jobs, evidence largely supports its effectiveness and superiority in comparison to other vocational rehabilitation interventions (de Winter et al., 2022; Frederick & VanderWeele, 2019; Richter & Hoffmann, 2019a; Suijkerbuijk et al., 2017). While SE leads to high rates of competitive employment, job retention and sustained work participation remain a challenge (McDowell et al., 2021). In contrast to SE, several workplace interventions exist that target employed individuals, aiming at preventing sick leave and fostering return to work (RTW). Available interventions are manifold and vocational support differs in content, structure, framework, professional qualification of providers and often include some type of psychological intervention such as cognitive behavioral therapy (McDowell et al., 2021; Nigatu et al., 2016; Proper & van Oostrom, 2019). Also, RTW studies often focus on individuals with common mental disorders (Nigatu et al., 2016). While various work-place programs show a positive effect on mental health and well-being (Proper & van Oostrom, 2019), beneficial effects on employment outcomes such as sick leave is less clear. A recent meta-analysis, including randomized-controlled trials with primarily psychological interventions found no evidence for enhanced RTW compared to care as usual (Nigatu et al., 2016). In contrast, some interventions such as workplace accommodation may improve job tenure (McDowell et al., 2021). Furthermore, there is still little known about SE based interventions supporting individuals at risk of job loss. Specifically, Telle et al., (2016) found no positive effect on sickness absence with SE in employed individuals. Duijts et al., (2008) found no evidence on sickness absence, using prevention coaching that has some similarity to SE.

With this current descriptive study, we aimed to contribute to the knowledge regarding the effectiveness of supported employment in supporting job maintenance in people with mental health problems, specifically mental illness ranging from moderate to severe impairments. We evaluated competitive employment proportions at time of discharge for SE in two distinct client groups: (a) individuals still employed (SE-retention), and (b) unemployed individuals (SE-integration). In order to understand the size of the difference in competitive employment proportions for SE in these two client groups, we made a descriptive comparison by using propensity score weighting. In contrast to SE-reintegration, SE-retention can be seen as early exclusion prevention that likely preserves important work-related resources. Thus, we hypothesized that SE-retention will show higher competitive employment proportions at discharge.

## Methods

### Research Type

This study was a single center, retrospective, descriptive, observational study based on administrative data provided by the Center for Psychiatric Rehabilitation (CPR), Universitäre Psychiatrische Dienste Bern (UPD), Switzerland. We used propensity score weighting to improve precision and reduce bias in treatment effects. This article was written in adherence with STROBE guidelines (von Elm et al., 2007). Classified as a quality measurement project, the local Swiss ethical board confirmed that the study was not subject to the Swiss Federal Act on research involving human beings (Req-2022-00235).

### Setting

The Bern Job Coach Placement is a CPR sub-department, which provides a wide range of vocational rehabilitation programs aimed at supporting employed or unemployed individuals with mental health problems and work-related issues to maintain/achieve competitive employment. Starting in early 2000 with supported employment based on individual placement and support (IPS) principles (Bond et al., 2020), various further vocational programs including job retention, supported education, and organisational interventions were developed and implemented. About 300 participants are supported yearly by a team of about 20 employment specialists experienced in vocational rehabilitation.

### Participants

The sample consisted of all individuals, aged ≥ 18 years at enrollment, with discharge between January, 2017 and October, 2021, that participated in either an SE-retention or an SE-reintegration program (described thereinafter), provided by the Bern Job Coach Placement. Generally, all individuals with mental health problems, that impaired their ability to work, were eligible to access the programs if funding was provided. Thus, participants with any mental illness ranging from moderate to severe impairments were eligible. Funding for vocational rehabilitation was usually provided by the Swiss Invalidity Insurance State Office or less frequently by other funders (e.g. employer, social welfare office). Although, most individuals who were admitted to vocational support were diagnosed with a ICD-10 psychiatric diagnosis, this was not a general prerequisite. The programs were also accessible for individuals with undiagnosed, self-perceived mental health problems that impedes their ability to work. External psychotherapeutic support was usually present or strongly recommended since this type of support was not covered by employment specialists. Individuals are in principle eligible for both programs described below (dependent on current employment status). It was also possible for individuals to receive SE-reintegration followed by SE-retention and vice versa. Further criteria for inclusion were: (a) a general ability to work, which is usually evaluated by the referring institutions in consultation with an involved physician; (b) wish to maintain/achieve competitive work; d) willingness/commitment to cooperate. Although not a specific exclusion criterion to access vocational programs, the Swiss Invalidity Insurance State Office does not usually provide funding for individuals with a recent substance abuse / use disorders and so these individuals were not included.

### SE-reintegration Program

The SE-reintegration program targets unemployed individuals and follows IPS principles with the exception of the zero exclusion criterion (Bond et al., 2020; Hoffmann et al., 2012). This is due to the funding conditions of the Swiss Invalidity Insurance, which requires that the funding institution may conduct eligibility assessments of potential participants. This program was already described and evaluated previously with a randomized controlled trial (Hoffmann et al., 2012, 2014). Employment specialists assist individuals in achieving and maintaining competitive employment on the basis of the participant’s educational background, work preference and previous work experience. If employment is achieved, the employment specialist provides on-the-job training and support individuals through regular contact (face-to-face, phone, email). Tasks and responsibilities of an employment specialist involve goal setting, evaluation of goal achievement, assessment of performance, support for conflict management and coping strategies, and work management skills. Special emphasis is placed on employer support and the involvement of funding agencies such as the invalidity insurance, close persons in the participant’s environment (e.g. relatives), and therapists. The length of participation was usually between 3 and 9 months.

### SE-retention Program

Similar to the SE-reintegration program, the SE-retention program is based on the same IPS core principles with a very similar type of support. In contrast, this program targets employed individuals at risk of job loss with the goal of maintaining competitive employment. Moreover, the content and focus can differ (e.g. job application support is not needed) and the individual support needs are typically lower. This program was initiated as an early intervention approach that preserves individual work-related resources and prevents labor market exclusion. Eligible participants are either sick on the job or on sick leave due to mental health problems and work-related issues. Eligible individuals either hold a permanent competitive job or are in a vocational/academic qualification process (supported education). Clients within the supported education program either hold an employment contract or are enrolled in a university. Length of participation is usually 3 months but can be extended to a much longer period as needed. In some cases, the supported education program assists individuals over several years.

### Measures

The single binary endpoint was success vs. non-success in the maintenance (SE-retention) or the achievement (SE-reintegration) of competitive employment. Individuals were considered successful if a temporary or permanent competitive employment contract was present at discharge. Competitive employment was defined as a regular job in the competitive labor market, that is open to any person, with at least minimal wage payment. In contrast, sheltered employment, referral to employment agency, early retirement, termination or withdrawal from the program were regarded as non-successes. Importantly, the status at discharge was the only available endpoint from the data source used.

Moreover, we used all available information on individual and program-related characteristics that were available in the data provided by the vocational service provider. Individual characteristics included gender (male, female), age at enrollment (years), nationality (Swiss, non-Swiss), main psychiatric ICD-10 diagnosis (was not available for all cases), presence of one or more secondary psychiatric diagnoses (yes, no), length of participation (days), year of discharge, and the number of previous vocational interventions received (categorized as 0, 1–2, 2–4, > 4). Importantly, individuals could participate repeatedly in any of the described programs. However, specific information on previous vocational interventions received was not available. Thus, cases were included only once in our data (dataset without repeated measures). There were no missing data except the specific main psychiatric ICD-10 diagnosis, which was not available for all participants.

### Statistical Analysis

Median and interquartile range (IQR) was reported for continuous variables and number and frequencies for categorical variables. Continuous and categorical characteristics between programs were compared using (unpaired) t-test/Wilcoxon and Chi-squared tests, respectively. For continuous variables, assumptions of normality and homogeneity of variances were tested using the Shapiro-Wilk-test and F-test of equal variances respectively. The Wilson method was used to calculate 95% confidence intervals (95%-CI) for proportions.

Logistic regression was used to descriptively compare SE programs and to investigate time trends. We reported odds ratios (OR) with associated 95%-CIs and predicted probabilities. To calculate predicted probabilities (i.e., the mean values) of competitive employment across years and for both programs (SE retention and reintegration), year by treatment interactions were used. Treatment effects (descriptive, competitive employment differences between programs) were calculated with: (a) a model with a treatment indicator only; and (b) a model with a treatment indicator using propensity score weights (PSW) (Rosenbaum & Rubin, 1983). PSW was used to improve the descriptive group comparison (reduce group differences). Weight estimation included all available characteristics which were potential confounders or outcome predictors (**Appendix 1** for details). Although, PSW improves the comparison, these two client groups remain partially comparable mainly due to their situation at admission (employed vs. unemployed). The statistical models were primarily used to understand the differences of SE in two distinct client groups regarding competitive employment and should be interpreted in a descriptive manner. All available cases were used for analysis and no prior power analysis was performed. Data analyses were conducted using R 3.5.0.33 mainly by using the survey package (Lumley, 2004).

## Results

### Participant Characteristics

Table 1 shows stratified characteristics by SE-retention (n = 297), SE-reintegration (n = 259), and the total of the combined programs (n = 556). The distribution of ICD-10 main diagnoses, the presence of a secondary diagnosis, and the number of previous vocational programs served in the past (prior to the current one) differed significantly across groups. Out of 297 within the SE-retention program, 13.1% (n = 39) participated in supported education and 86.9% (n = 258) in regular job retention support.

**Table 1:** Participant characteristics by SE-retention and SE-reintegration program and total group

Characteristic	SE-retention	SE-integration	Total	Group comparison (p-value)
	(N = 297)	(N = 259)	(N = 556)	
Gender				p = 0.64^a^
Male	132 (44.4%)	109 (42.1%)	241 (43.3%)	
Female	165 (55.6%)	150 (57.9%)	315 (56.7%)	
Age (years)				p = 0.22^b^
Median (IQR)	43.2 (22.5)	39.3 (15.5)	41.0 (20.0)	
Min.; max.	18.1;63.8	18.0;62.9	18.1;63.8	
Nationality				p = 0.12^c^
Swiss	294 (99.0%)	251 (96.9%)	545 (98.0%)	
Non-Swiss	3 (1.0%)	8 (3.1%)	11 (2.0%)	
ICD-10 Diagnoses				p = 0.04^c^
Other^d^	31 (10.4%)	17 (6.6%)	48 (8.6%)	
F2, Schizophrenia	19 (6.4%)	24 (9.3%)	43 (7.7%)	
F3, mood disorders	176 (59.3%)	137 (52.9%)	313 (56.3%)	
F4/5, neurotic disorders & behavioral syndromes	36 (12.1%)	35 (13.5%)	71 (12.8%)	
F6, personality disorders	12 (4.0%)	25 (9.7%)	37 (6.7%)	
F8/F9, disorders childhood/adolescence	23 (7.7%)	21 (8.1%)	44 (7.9%)	
Secondary Diagnosis				p = 0.017^a^
Yes	109 (36.7%)	122 (47.1%)	231 (41.5%)	
No	188 (63.3%)	137 (52.9%)	325 (58.5%)	
Length of participation (days)				p = 0.76^b^
Median (IQR)	111 (121)	104 (115)	107 (120)	
No. previous vocational interventions				p < 0.001^a^
None	155 (52.2%)	52 (20.1%)	207 (37.2%)	
1–2	83 (27.9%)	104 (40.2%)	187 (33.6%)	
3–4	37 (12.5%)	69 (26.6%)	106 (19.1%)	
>4	22 (7.4%)	34 (13.1%)	56 (10.1%)	
Year of intervention				p = 0.37^a^
2017	42 (14.1%)	52 (20.1%)	94 (16.9%)	
2018	50 (16.8%)	38 (14.7%)	88 (15.8%)	
2019	66 (22.2%)	49 (18.9%)	115 (20.7%)	
2020	63 (21.2%)	58 (22.4%)	121 (21.8%)	
2021	76 (25.6%)	62 (23.9%)	138 (24.8%)	

^a^ Chi Squared test

^b^ Wilcoxon test (unpaired)

^c^ Fisher’s exact test

^d^ Other includes (total sample); F1 substance use (N = 3), F7 mental retardation (N = 5), and unknown or F99 unspecified diagnoses (N = 48)

Abbreviations: SE-retention = Supported employment job retention program; SE-reintegration = Supported employment job reintegration program

### Competitive Employment over time

**Fig. 1:**
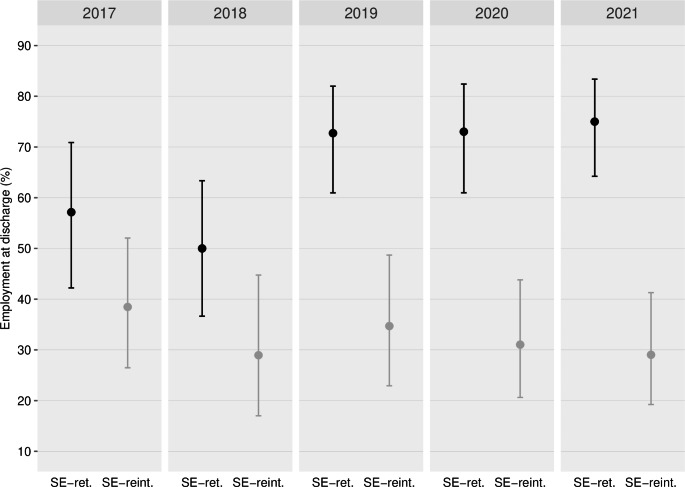
Predicted probabilities (%) of achieving competitive employment for SE-retention and SE-reintegration by year of discharge. Shown are predicted probabilities (mean and 95% CI) for each program and by year of discharge. Predicted probabilities were calculated using unadjusted logistic regression with time by treatment interaction without further covariates. Abbreviations: SE-ret. = Supported employment job retention program; SE-reint. = Supported employment job reintegration program

Figure 1 shows the unadjusted predicted competitive employment probabilities (95%-CIs) for SE-retention (black bars) and SE-reintegration (gray bars) by year. Descriptively, the SE-retention showed an increase, while SE-reintegration showed a decline over time. For SE-retention, employment proportions were significantly higher in more recent years as compared to earlier years (e.g. higher proportions for years 2019–2021 as compared to 2017 or 2018). For the SE-reintegration, proportions did not significantly differ between years. Figure 1**appendix** provides further information on SE-retention sub-programs and SE-integration showing yearly employment proportions and case numbers by year. Within the SE-retention program, supported education was descriptively somewhat less successful than usual job retention.

### Descriptive Comparison of SE-retention and SE-reintegration

Table 2 shows the treatment effect and estimated predicted probabilities. The descriptive group comparison (treatment effects) yielded an unadjusted OR of 4.30 [95% CI: 3.02 to 6.15, p < 0.001] favoring SE-retention with associated predicted probabilities of maintaining/achieving competitive employment of 67.3% and 32.4% for SE-retention and SE-reintegration, respectively. Similar results were found for the adjusted model with propensity score weighing with an OR of 4.85 [95% CI: 3.10 to 7.58, p < 0.001] comparing SE-retention to SE-reintegration.

**Table 2:** Comparison of SE-retention with SE-integration with regard to competitive employment at discharge

Model	Odds Ratio (95%-CI)	p-value	predicted probability (%)
Unadjusted treatment effect ^a^			
SE-reintegration	Reference		32.4
SE-retention	4.30 (3.02 to 6.15)	< 0.001	67.3
Weighted treatment effects ^b^			
SE-reintegration	Reference		29.9
SE-retention	4.85 (3.10 to 7.58)	< 0.001	67.3

^a^ logistic regression with treatment indicator only (without any covariate adjustment)

^b^ Average treatment effect on the treated from inverse probability weighted logistic regression

Abbreviations: SE-retention = Supported employment job retention program; SE-reintegration = Supported employment job reintegration program

## Discussion

In this descriptive study we evaluated and compared the competitive employment proportions of an SE based vocational program in two distinct groups including employed and unemployed individuals with mental health problems. Not surprisingly, the weighted analysis revealed that SE-retention participants were substantially more likely to be employed at discharge as compared with the SE-reintegration program (67% vs. 30%). While SE-retention became increasingly successful in recent years (unadjusted trend), the employment proportions for SE-reintegration remained approximately stable. Although, this evaluation is limited by its descriptive nature and comparison of two distinct groups, the results suggest that job retention programs based on IPS may be a promising approach to prevent vocational exclusion.

The descriptive results showed better outcomes in SE-retention compared to SE-reintegration. Although, we adjusted the analyses for some relevant factors, the differences found are only partially attributable to differences in vocational treatments. Given the expected differences in characteristics between the two client groups in our study, the propensity score weighing was used to improve the comparability as much as possible. Standardized mean differences suggested a sufficient balance of included covariates such as age (see Appendix). However, while individuals allocated to SE-retention were still employed, participants of SE-reintegration were unemployed at admission. Given this, it is plausible to expect higher competitive employment proportions at discharge for SE-retention clients compared to SE-reintegration. The employment status was part of the inclusion criteria for the study, and therefore influenced employment outcomes by design and also acted as a confounder due to its effects on the group comparisons.

We were also unable to evaluate the comparability between the groups regarding the illness severity, which can be considered an important influential factor as employment rates are known to decrease with increasing mental illness severity (Luciano & Meara, 2014; Richter & Hoffmann, 2019b). Employment and mental health show bi-directional associations; poor mental health is a risk factor of and a risk factor for unemployment (Olesen et al., 2013). Furthermore, the recent work history is strongly associated with the achievement of employment (Metcalfe et al., 2017). SE-reintegration participants are likely to be exposed to much less favorable conditions leading to a lower likelihood of employment and further disadvantages such as poverty, lack of social support, and lower educational levels (Etuknwa et al., 2019; Richter & Hoffmann, 2019b).

The findings suggested an increase in employment proportions over time for the SE-retention but not for the SE-reintegration program. Based on the administrative data used, we were not able to further investigate these trends. It is conceivable that variations may be explained by several factors including, participant characteristics, labor market conditions, changing circumstances due to the Corona pandemic, alterations in financing practices of funding agencies, an increase in experience by the vocational institution in supporting employed individuals or relevant staff fluctuations.

Comparing the results for the SE-retention program with current research is generally difficult due to several reasons. First, SE-retention generally includes both individuals with or without sick leave due to mental health problems and work-related issues. As this is a different starting point for intervention, the expected clinical and work-related outcomes may differ. Second, SE-retention provides support for academic/vocational education but also assistance for regular jobs. As shown descriptively, success was somewhat lower for supported education compared to the classical job retention sub-program. However, contrast for sub-programs were not evaluated since very little data were available for supported education. Third, other studies usually use different work-related outcomes like sickness absence, job tenure, performance/skills, functional or clinical outcomes.

Our results are, however, approximately comparable with a meta-analysis that looked at effects of mainly psychological interventions in individuals with common mental disorders on the RTW proportion. Nigatu et al., (2016) found pooled rates of 65% for intervention and 60% for treatment as usual, which did not differ significantly. In a randomized controlled trial, Reme et al., (2015) investigated the effect on work participation of a work-focused program with cognitive behavioral therapy and IPS in participants with common mental disorders. Work participation was 44.2% in the intervention group at the 12-month follow-up (37.2% in controls), which is lower than the proportions found in our study. This discrepancy may be explained by the investigation of different client groups including individuals on long-term benefits. Telle et al., (2016) conducted a randomized-controlled trial with a similar SE-retention intervention which showed a positive effect on secondary outcomes like depressive symptoms but no effect on sickness absence days. Duijts et al., (2008) were not able to detect a difference in sickness absence with preventative coaching, that shares some similarities with SE-retention coaching, for people at risk of sickness absence due to psychological health complaints.

The competitive employment proportions found for SE-reintegration (31%) is comparable to 33% found in an earlier evaluation of the same program (Richter et al., 2019). Except for two studies showing more extreme results (9 and 54%), employment proportions at discharge found in this study are in the range of previous studies that found employment proportions between 25 and 43% at the end of various follow-up time points (Frederick & VanderWeele, 2019). In contrast, results are lower than pooled competitive employment rates of 43% found in a meta-analysis of non-trial routine programs or optimal quarterly performance benchmarks of 45% (Becker et al., 2011; Richter & Hoffmann, 2019a). The likely reason for this substantial divergence is the difference in outcome operationalization. In contrast to the outcome used in this study (job contract at discharge), competitive employment rate is usually defined as the proportion of participants that held a competitive job (≥ 1 day) within a defined time interval (Becker et al., 2011; Gold et al., 2010). Thus, in this study competitive employment was operationalized more conservatively and reflect a regular transition to the competitive labor market.

### Practical Implications and Further Directions

Results for the SE-retention are promising and suggest that early support with work-related issues is key to reduce exclusion from the competitive labor market. Once unemployed, considerably more effort may be needed for reintegration. Yet, the majority of SE-reintegration participants were not successful regarding a transition to the regular job market. Competitive employment rates achieved, using a frequently used definition (Bond et al., 2012), are often remarkable across studies but suffer the limitation of not reflecting a transition to the competitive labor market. Moreover, job tenure can be relatively brief, performance can be low for people with high support needs, and positive effects can decrease over time in SE-reintegration programs (McDowell et al., 2021; Pichler et al., 2021). The effectiveness of vocational programs is generally influenced by multiple factors, some of which cannot be altered like local environmental factors (Metcalfe et al., 2018). With regards to modifiable factors, it seems to be important to understand how such programs can be adapted to changes in the labor market and which augmentations work best to maximize outcomes. Augmentations like skills training, access to further education but also social support, employment specialist practices and other factors should be further considered for developing IPS based vocational programs (Dewa et al., 2018).

### Strengths and Limitations

A strength of our study is that this is a large observational study covering a five-year time-span that allowed for us to look at variability over time. To improve the descriptive comparison, some important confounders potentially related to program assignment and the outcome were taken into account. The two programs evaluated are well established and led by a specialized institution with experienced employment specialists and access to professional psychological support. Limitations arise from the observational nature of the study design, the use of administrative data not gathered for research purposes, and unobserved confounders. Moreover, SE-reintegration as comparative condition for SE-retention is strongly limited as groups likely differ in many ways beyond treatment, such as the employment status at program admission (employed vs. unemployed). From a theoretical point of view, there are also many other factors potentially influence treatment assignment and outcome jointly such as social skills, educational background, work experience, the clinical history, and the severity of the mental health problem all of which were not included in our analysis. Nevertheless, we aim to highlight the higher success rate of an SE-retention program compared to SE-reintegration to show that support for these individuals should ideally come before they quit or otherwise lose/leave their job. Although, our outcome measure reflects a regular competitive labor market transition, there were no follow-up data available which precludes statements regarding job tenure or competitive employment that was achieved shortly after discharge. Our analysis was further restricted by low case numbers (supported education), missing group identifiers (occurrence of sick leave), and the unavailability of further information (e.g. sick-leave days).

## Conclusion

In this study we compared an SE vocational intervention in two distinct groups. SE based job retention support may be a largely promising approach in preventing vocational exclusion in employed individuals with mental health problems at risk of job loss. Yet, reintegration in unemployed individuals is likely to require more efforts to achieve competitive employment, likely with less favorable outcomes. In our study, the majority of the SE-retention participants were able to remain employed. In contrast, only the minority of SE-reintegration participants who were unemployed at admission achieved competitive employment. Although, our descriptive comparison of the same intervention in two distinct groups included several limitations, early access to SE for those still employed may preserve individual resources that can be key to improve competitive employment. Consequently, early detection of those in need and appropriate access to work-related support can be crucial. SE programs to maintain employment should be further developed and emphasis should be placed on clearly defining the content of these support programs to further enhance the likelihood of job retention. Studies with more rigorous designs, incorporating appropriate control conditions, are needed to further investigate treatment effects, personal and environmental factors, coaching regimes and intensity, and longer-term outcomes.

## Electronic Supplementary Material

Below is the link to the electronic supplementary material.

Supplementary Material 1

## Declarations

This manuscript was written in accordance to STROBE guidelines (The Strengthening the Reporting of Observational Studies in Epidemiology Statement: guidelines for reporting observational studies).
