# Supplementary material for: Job Retention and Reintegration in People with Mental Health Problems: A Descriptive Evaluation of Supported Employment Routine Programs
Source: Adm Policy Ment Health. 2022 Oct 26;50(1):128–36. doi: 10.1007/s10488-022-01227-w (PMC9832069; doi:10.1007/s10488-022-01227-w)
Supplement: Supplementary file 1 — Supplementary Material 1 [file 10488_2022_1227_MOESM1_ESM.docx]

# SUPPLEMENTARY MATERIAL

## Appendix 1

Propensity score weights were calculated using logistic regression without interactions or higher order terms. The model included gender, age, main psychiatric ICD diagnostic category, secondary psychiatric ICD diagnosis, the number of previous vocational rehabilitation programs received, and year of discharge coded as described in the manuscript. Nationality was not included in this model due to low case numbers with non-Swiss origin. Also, the length of participation was not included since this variable was measured after program entry and potentially qualifies as mediator. Therefore, based on theoretical considerations, variables included were either assumed to influence the outcome (competitive employment) or the probability of treatment assignment and outcome (confounder).

The evaluation of common support (comparing the distribution of propensity scores separated by group) was evaluated using graphical tools. We used the weighting by the odds approach to estimate the average treatment effect on the treated (ATT) (Harder, Stuart, & Anthony, 2010). No truncation of weights was done since extreme weights were absent. Covariate balance was investigated comparing weighed means of the two treatment groups (SE-retention and SE-reintegration). Standardized mean differences suggested a sufficient balance of covariates. Appendix Table 1 shows the weighted means for each group and the p-value associated with the comparison by the t-statistic or chi-square statistic for numeric and factor variables, respectively.

| **Appendix Table 1**  Description of covariate balance comparing weighted means across treatment groups (SE-retention, SE-reintegration) | | | | |
| --- | --- | --- | --- | --- |
| Covariate | weighted means SE-retention | weighted means SE-reintegration | Standardized effect size | p-value |
|  |  |  |  |  |
| Male | 0.44 | 0.43 | 0.03 | 0.7391 |
| Female | 0.56 | 0.57 | -0.03 |  |
| Age (years) | 41.10 | 41.10 | 0.00 | 0.5750 |
| ICD-10 diagnosis: Other | 0.10 | 0.12 | -0.04 | 0.9908 |
| ICD-10 diagnosis: F2, Schizophrenia | 0.06 | 0.06 | 0.02 |  |
| ICD-10 diagnosis: F3, mood disorders | 0.59 | 0.57 | 0.04 |  |
| ICD-10 diagnosis: F4/5, neurotic disorders & behavioral syndromes | 0.12 | 0.12 | 0.02 |  |
| ICD-10 diagnosis: F6, personality disorders | 0.04 | 0.05 | -0.04 |  |
| ICD-10 diagnosis: F8/F9, disorders childhood/adolescence | 0.08 | 0.09 | -0.03 |  |
| Secondary ICD diagnosis: Yes | 0.37 | 0.36 | 0.02 | 0.8569 |
| Secondary ICD diagnosis: No | 0.63 | 0.64 | -0.02 |  |
| Previous interventions; 0 | 0.52 | 0.52 | 0.01 | 0.9971 |
| Previous interventions; 1-2 | 0.28 | 0.28 | -0.01 |  |
| Previous interventions; 3-4 | 0.12 | 0.13 | -0.01 |  |
| Previous interventions; >4 | 0.07 | 0.07 | 0.01 |  |
| Year: 2017 | 0.14 | 0.16 | -0.05 | 0.9309 |
| Year: 2018 | 0.17 | 0.18 | -0.03 |  |
| Year: 2019 | 0.22 | 0.20 | 0.05 |  |
| Year: 2020 | 0.21 | 0.19 | 0.06 |  |
| Year: 2021 | 0.26 | 0.27 | -0.04 |  |

## Figure 1 appendix

Probabilities (%) of achieving competitive employment and case numbers by year for SE-retention sub-programs and

SE-reintegration, respectively


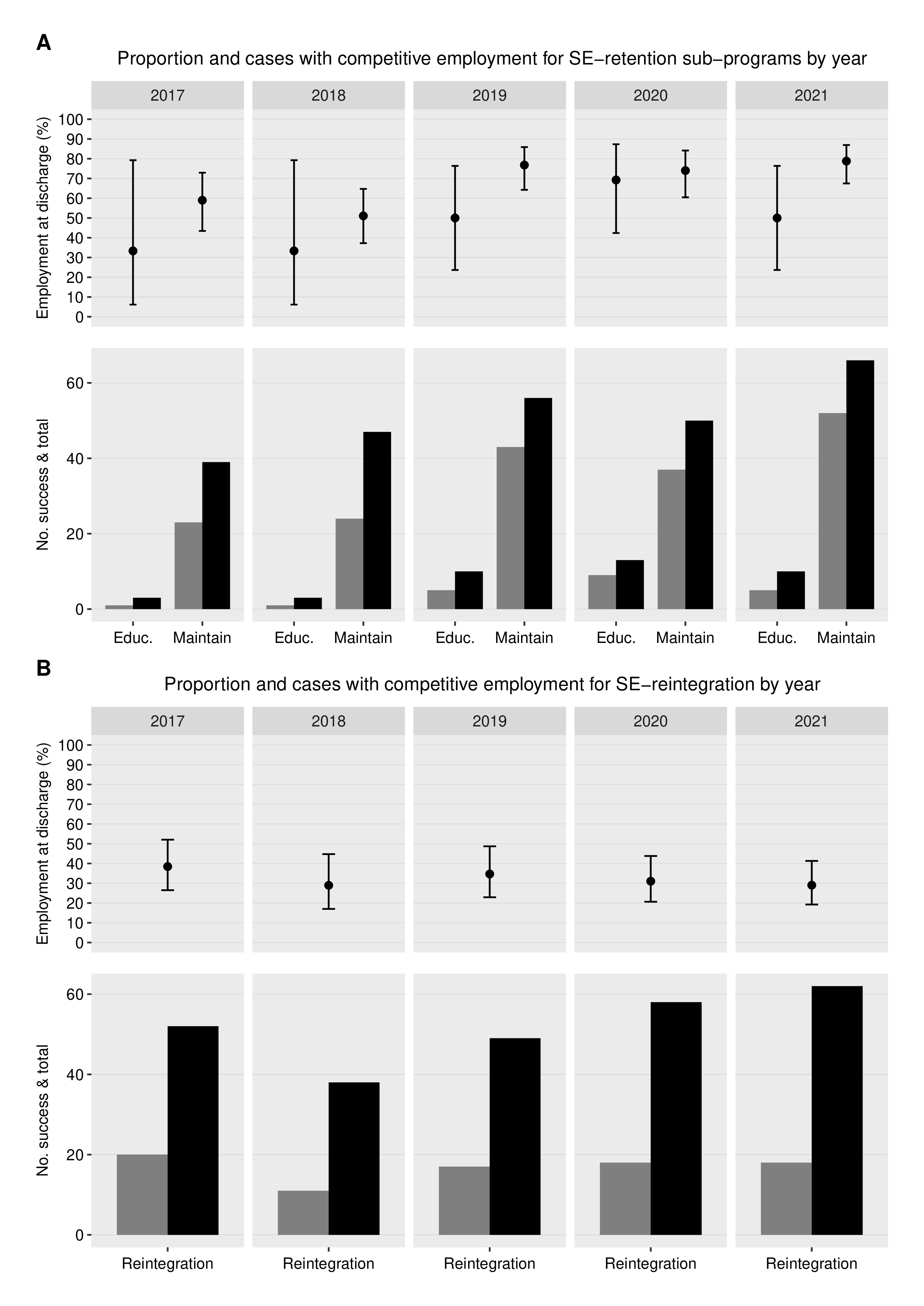


**Figure 1 appendix caption:** Plot A shows SE-retention subprograms including support during vocational education (Educ.) and support for individuals with a permanent job (Maintain). Plot B shows SE reintegration program. The dot charts show the mean proportions [including 95%-Wilson CI, by year and (sub-)program]. The bar charts show the number of individuals with competive employment at discharge (gray bar) and the total number (black bar) of participants by year.

## Appendix references

Harder, V. S., Stuart, E. A., & Anthony, J. C. (2010). Propensity score techniques and the assessment of measured covariate balance to test causal associations in psychological research. *Psychol Methods, 15*(3), 234-249. doi:10.1037/a0019623
